# Exploring potential barriers in equitable access to pediatric diagnostic imaging using machine learning

**DOI:** 10.3389/fpubh.2023.968319

**Published:** 2023-02-24

**Authors:** Maryam Taheri-Shirazi, Khashayar Namdar, Kelvin Ling, Karima Karmali, Melissa D. McCradden, Wayne Lee, Farzad Khalvati

**Affiliations:** ^1^Department of Diagnostic Imaging, The Hospital for Sick Children (SickKids), Toronto, ON, Canada; ^2^Institute of Medical Science, University of Toronto, Toronto, ON, Canada; ^3^Vector Institute, Toronto, ON, Canada; ^4^NVIDIA Deep Learning Institute, Austin, TX, United States; ^5^Department of Bioethics, The Hospital for Sick Children (SickKids), Toronto, ON, Canada; ^6^Peter Giligan Centre for Research and Learning - Genetics and Genome Biology Program, Toronto, ON, Canada; ^7^Dalla Lana School of Public Health, University of Toronto, Toronto, ON, Canada; ^8^Department of Medical Imaging, University of Toronto, Toronto, ON, Canada; ^9^Department of Mechanical and Industrial Engineering, University of Toronto, Toronto, ON, Canada; ^10^Department of Computer Science, University of Toronto, Toronto, ON, Canada

**Keywords:** appointment scheduling, no-show, waiting room time, logistic regression, random forest

## Abstract

In this work, we examine magnetic resonance imaging (MRI) and ultrasound (US) appointments at the Diagnostic Imaging (DI) department of a pediatric hospital to discover possible relationships between selected patient features and no-show or long waiting room time endpoints. The chosen features include age, sex, income, distance from the hospital, percentage of non-English speakers in a postal code, percentage of single caregivers in a postal code, appointment time slot (morning, afternoon, evening), and day of the week (Monday to Sunday). We trained univariate Logistic Regression (LR) models using the training sets and identified predictive (significant) features that remained significant in the test sets. We also implemented multivariate Random Forest (RF) models to predict the endpoints. We achieved Area Under the Receiver Operating Characteristic Curve (AUC) of 0.82 and 0.73 for predicting no-show and long waiting room time endpoints, respectively. The univariate LR analysis on DI appointments uncovered the effect of the time of appointment during the day/week, and patients' demographics such as income and the number of caregivers on the no-shows and long waiting room time endpoints. For predicting no-show, we found age, time slot, and percentage of single caregiver to be the most critical contributors. Age, distance, and percentage of non-English speakers were the most important features for our long waiting room time prediction models. We found no sex discrimination among the scheduled pediatric DI appointments. Nonetheless, inequities based on patient features such as low income and language barrier did exist.

## 1. Introduction

The noble goal of medicine is to provide compassionate healthcare to all persons, regardless of ethnicity, race, sex, age, and socioeconomic status. Healthcare delivery is notably affected by implicit bias (1), accessibility barriers (2–4), and systemic racism reflecting structurally unequal patterns of housing, education, and policing. Despite all the advancements in healthcare in the past decades, disparities based on race and ethnicity persist in access to healthcare, the quality of care received, life expectancy, and mortality. Subtle biases and implicit attitudes often exist out of conscious awareness, and therefore, it makes it difficult to acknowledge and control. As such, healthcare requires a more explicit commitment to the ethical principle of equity, which entails proactive and targeted decisions that advance the interests of those who are the least advantaged and are under-represented in the healthcare system. To reduce the racial and ethnic disparity and provide care in a manner that compensates for the relative lack of privileges, we must first characterize the discrepancy in access patterns to identify opportunities to take equity-promoting actions in healthcare (3).

As The Hospital for Sick Children (SickKids) adopts a digital-first strategy to enhance care delivery, a careful eye to equity can enable us to better deliver care to those who need it the most. Biases are complex and multi-faceted, and our project takes aim at the issue of accessibility for Diagnostic Imaging (DI) procedures. The first step toward enhancing equitable care is by empirically studying the current patterns in access to detect systematic barriers that our patients and families face.

In the department of DI at SickKids in Canada, approximately 3,000 appointments are scheduled every month. DI is an important step in disease diagnosis where treatment path is often planned. Depending on their health issue, patients might be referred to different modalities of diagnostic imaging, such as magnetic resonance imaging (MRI) or ultrasound (US). Based on the results of DI reported by the radiologists, the next step in patient care is decided. This may include additional tests (e.g., blood tests), tissue biopsy, or start of treatment. Timely access to DI appointments is crucial for optimal care delivery to patients. Thus, it is vital to identify whether there is any bias when the appointments are assigned to the patients and if so, to ensure equitable access to all patients to DI appointments, with the available medical imaging resources at the DI.

To investigate the potential impact of equity-related patient identifiers and access to DI appointments at the hospital, we collected, curated, and analyzed appointment data of 42,795 unique patients admitted to DI during 2018–2021. The dataset included MRI and US appointments. The selected features from the datasets were age, sex, income, distance from the hospital, percentage of non-English speakers in a postal code, percentage of single caregivers in a postal code, and appointment time slot (morning, afternoon, evening), and day of the week (Monday to Sunday). The main objective is to investigate the relationships between selected patient features and no-show and waiting room time for more than 1 h endpoints.

We applied univariate Logistic Regression (LR) and reported the Odds Ratio (OR) and *p*-values of the features with the two endpoints, no-show, and waiting room times for more than 1 h. We also implemented multivariate Random Forest models (RF) to predict the two endpoints. The organization of this paper is as follows: a brief literature review of related work is presented in Section 1. Section 2 presents the dataset and explains the methods followed by results shown in Section 3. Sections 4 provides the discussion.

The main contributions of this paper include the followings:

- We curated a large dataset of appointments from the diagnostic imaging department of the SickKids hospital with over 74,000 entries.
- We conducted modality-specific as well as overall studies.
- We augmented the dataset through adding important features from the Canada Statistics Census such as percentage of single caregivers in neighborhoods.
- We conducted feature transformation and statistical analysis to find the best representation of each feature before feeding them into the machine learning models.

### 1.1. Literature review

The study of no-shows and waiting room times and corresponding predictive models are usually distinct topics in the literature. Hence, in the following, the relevant papers are reviewed separately.

#### 1.1.1. No-show

During the last decades, a significant number of experiments have been conducted to analyze no-show endpoints and search for solutions to predict and mitigate the consequences of these endpoints *via* analytical techniques (5). No-show appointments, when the patients miss their scheduled appointments without notifying the healthcare provider, cause significant impacts on revenue, cost, and use of resources in healthcare systems (6). No-show specifically for diagnostic imaging can negatively affect the patients' health. No-shows among patients are not arbitrary, and arise out of situational factors (e.g., patient behaviors, extenuating circumstances, accessibility barriers) (7, 8). The statistical analysis of this relationship led to implementation of multiple statistical techniques to reduce the negative effects of no-show appointments, namely, overbooking (9–12), open-access scheduling (13), or using fines to penalize those who miss their appointment (14).

Studies show that the no-show rates may vary from 3 to 80% depending on the patient population, type of clinic, the continent where the study was performed, the year the study was conducted, and the medical specialty (6). Different statistical analyses, including univariate and multivariate analyses, have been used for studying no-show. Logistic Regression (LR) (binary and multinomial LR) are the most common methods in the literature to predict the no-show appointments (5, 9, 13, 15–18).

Huang et al. (19) used a dataset of about 7,000 unique patients and developed several LR models based on the number of patients' visits. There were 26 predictive models developed based on the number of available past appointments. Models with a higher number of patients' produced more accurate results. The maximum area under the receiver operating characteristic curve (AUC) was 0.706, which was for the model that used patients' data who had at least 19 visits.

Kurasawa et al. (20) reported achieving AUC of 0.958 with an LR model on University of Tokyo Hospital data, which included about 16,000 appointments scheduled for 879 unique patients, with the inclusion of important predictors such as patient's clinical condition, department, disease, and prescribed medicine. They also included other characteristics such as sex, age, distance from the hospital, frequency of clinic visit, probability of visit on a given day of the week, interval between the scheduling date and appointment date, day of the week, previous no-show, weather, length of prescription, the total amount of medicine per day, how many times a day a medication is taken, and maximum size of prescribed tablets.

Lin et al. (21) proposed LR models with Bayesian Lasso for feature selection and achieved AUC between 0.70 and 0.92 for 475 providers (doctors) grouped by 53 clinics, containing 1,000 to 21,000 patients each, with an average of 4,404 patients. Several works studied the advantages of using decision trees (DTs) compared to LR. For instance, Devasahay et al. (22) achieved a reasonable specificity (99.3%) but with inferior sensitivity (3.5%) compared to LR with specificity and sensitivity of 99.9 and 0.1%, respectively. Neural Network (NN) methods which are currently receiving a high level of attention in the field of artificial intelligence, have also been used for studying no-show. For instance, Aladeemy et al. (23) developed an optimization algorithm called integration of Self-Adhesive Cohort Intelligence with opposition-based learning strategies, and its performance was compared against that of Genetic Algorithms (Particle Swarm Optimization, Differential Evolution), as well as RF, Ada Boost, implemented Support Vector Machine (SVM), Naïve Bays (NB), K-nearest neighborhood (KNN), Deep Neural Network (DNN), and Elastic-Net Regularized Generalized Linear model, achieving 0.72 AUC, 0.81 sensitivity, and 0.61 specificity. Dashtban and Li (24) developed a sparse stacked denoising autoencoder for no-show prediction. The proposed auto-encoder was trained with a database of 1.6 million appointments, achieving an AUC and accuracy of 0.71, 0.69, respectively. Mohammadi et al. (25) implemented the Naïve Bayes methods along with the LR and NN on 74,000 unique appointments, and achieved up to 0.90 AUC.

Different features and predictors have been used in no-show analysis, including age, sex, race, socioeconomic status, and level of education. Most studies showed that the no-show rate has an inverse relationship to the age of the patients, meaning that young adults are most likely to miss their appointment. Multiple studies confirmed sex is not a statistically significant predictor of no-show, but a few studies reported that men were more likely to miss their appointments than women (26). Moreover, members of minority groups across countries tend to have higher rates of no-show. Lower economic level and marital status are other factors in no-show rate. There is an inverse relationship between income and likelihood of no-show across studies. While marital status seems to be a less predictive feature, a few studies showed that being a single caregiver increases the probability of no-show (6, 27). For pediatric appointments, a lower parental educational level was associated with increased no-show behavior. There are a few other factors such as the lead time, the interval between the time when the patient schedules the appointment and the actual appointment time, prior no-show history, date and time of the appointment, source of referral, type of visit, and the number of previously scheduled visits. It was found that the lead time and prior no-show were the most important predictors of no-show (28). Other features and predictors such as day of the week, month of appointment, and appointment time were also found to be insignificant features of no-show.

#### 1.1.2. Waiting room time

Patients who keep their appointments may experience negative effects, including dissatisfaction with high waiting room time and service quality (29). However, Anderson et al. (30) used a web-based survey on 5,000 patients and showed that time spent with the doctor is more influential than the waiting room time. Sun et al. (31) predicted the emergency department (ED) waiting room time by quantile regression models combined with queue length for more accuracy. They also provided a range from median to the 90th percentiles for the waiting room time to compensate for the inaccurate median waiting room time. In their validation, the median absolute prediction error was 9.2 min for patients with priority type 2 (the patient experiences severe, difficult to manage symptoms which are likely getting worse) and 12.9 min for patients with priority type 3 (the patient experiences some pain or other symptoms which do not dramatically impact the quality of life). Bell et al. (32) used time series models such as auto-regression integrated with moving average and autoregressive integrated moving average (ARIMA) errors to capture the short-term fluctuation better.

Huang et al. (33) proposed a hybrid model that combines the ARIMA errors with adaptive filtering, achieving a higher accuracy (up to 0.88 to 0.99 improvement) than the traditional ARIMA models. Ang et al. (34) used the number of patients waiting in the ED to start the treatment, the number of providers in ED, the rate the provider treats the low-acuity patients, and the total processing rate for low-acuity patients as independent variables to achieve 30% lower mean square error (MSE) with Q-Lasso models compared to MSE of moving average methods.

Guédon et al. (35) developed a real-time predication system to classify surgeries into two categories; surgeries that are shorter or longer than a specific time. They SVM on the data retrieved from the medical devices. Arha (36) used quantile and regularized regression such as Lasso, Ridge, Elastic Net, Smoothly Clipped Absolute Deviation (SCAD), Minimax Concave Penalty (MCP) through mean square error, and RF to predict patient's waiting room time. Queue of patients at different stages of ED and patient's arrival time (days, week, and month) were used as predictors. Among all methods, RF had the highest accuracy.

Chen et al. (37) predicted the waiting room time for each treatment in the hospital, and they developed a recommendation system for patient's treatment plan based on the predicted time. Patients would see the plan and expected waiting room time for each treatment in real-time. They used the cloud implementation of RF to handle the scalability and efficiency of the model. They utilized patient's sex, age, department, doctor's name, task's name, start time, end time, week, treatment time, and the time interval between appointments as their predictors and achieved an accuracy above 0.92.

Gonçalves et al. (38) used RF to predict the category of the emergency waiting room time, the categories they used in their analysis are from “really low” to “really high”, they achieved an accuracy of 50%, which is not a reliable performance. Kuo et al. (39) implemented machine learning methods for real-time and personalized waiting room time prediction, including stepwise linear regression, artificial neural network, SVM, and gradient boosting machines. They achieved 17–22% reduction in mean square error compared to simple linear regression as baseline method, suggesting the machine learning methods can improve the performance of waiting room time prediction.

## 2. Materials and methods

### 2.1. Dataset

**Figure 1:**
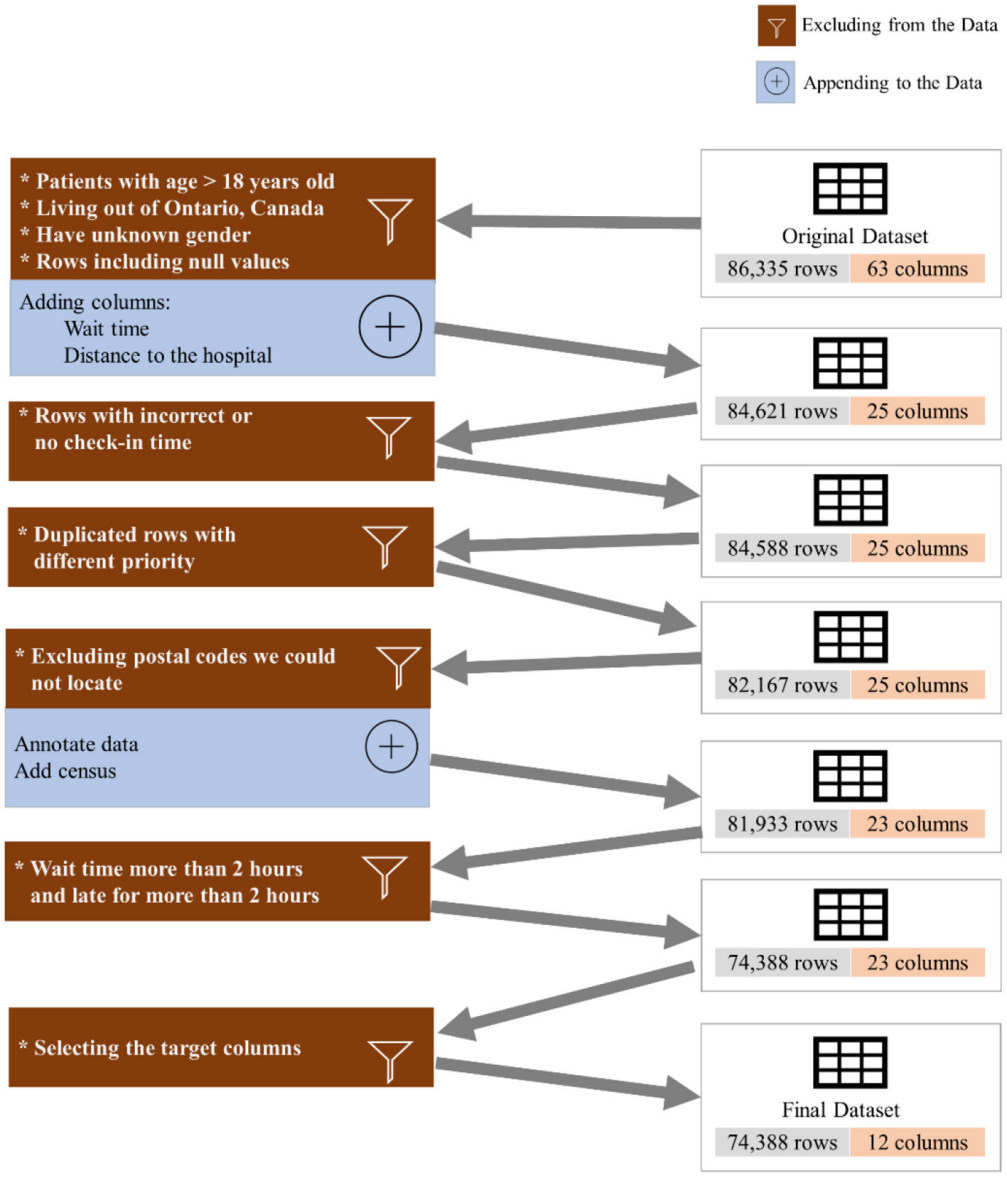
Data pre-processing.

To investigate the existence of inequality in access to DI services, we collected and analyzed 86,335 appointments of 42,795 unique patients from June 2018 to March 2021 (34 months) from the DI department at the SickKids Hospital. It should be highlighted that SickKids, in the context of appointment scheduling, considers caregivers, patients and the family as a unit, and thus the term “family” could be used as an alternative to “patient” if we were not conforming literature terminology. Figure 1 shows the inclusion/exclusion criteria and the process of cleaning the dataset. We excluded patients above 18 years old, those who lived outside Ontario, and anyone with missing features or incorrect check-in times. Additionally, we appended new features from the Canada Statistics Census^1^ and dropped all but the target features. Table 1 shows the features that we used in our experiment, along with their full descriptions. The features include age, sex, distance to the hospital based on the postal code, average family income in a postal code, percentage of the single caregiver in a postal code, percentage of the non-English speaking caregiver in a postal code, time of appointment, and day of appointment within the week (weekday).

**Table 1:** Description of features used in our experiment.

**Feature**	**Description**
Income	An estimation of the household income based on their postal code, extracted from the Canada Statistics Census
Distance	An estimation of the distance from patient's postal code to the SickKids Hospital's postal code. We used the “pgeocode^a^” package in Python to get the estimated longitude and latitude of the patient's and the hospital postal code and then calculate the distance between the two points using the Haversine formula.
% Non-english speaker	An estimation of the non-English speaking residence percentage in a postal code extracted from the Canada Statistics Census
% Single caregiver	An estimation of the single caregiver percentage in a postal code extracted from the Canada Statistics Census.
Age	Age of the patients at scan
Time slot	Time of the patient's appointment has been categorized into 3 categories: morning (up to 12 pm), afternoon (12–6 pm) and evening (after 6 pm)
Sex	Sex assigned on patient's health card
Weekday	Monday to sunday

^a^https://pypi.org/project/pgeocode/.

We eliminated patients with age higher than 18 years old, individuals who live outside of Province of Ontario, Canada, those who reported no sex, anyone with incorrect or no check-in time, patients who waited for more than 2 h or were late for more than 2 h, and we excluded the duplicated rows and null columns. This resulted in 74,388 appointments. We reported the result of our experiment for three different datasets: (a) the whole dataset (All), which contains all imaging modalities, such as MRI, US, and Computed Tomography (CT), Magnetoencephelography (MEG), and Image Guided Therapy (IGT) scan appointments, (b) a dataset containing only US appointments, and (c) a dataset containing only MRI appointments. Table 2 shows the distribution of the endpoints for each dataset.

**Table 2:** Distribution of endpoints.

** 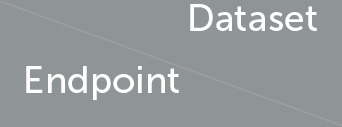 **	**All (the whole dataset)**	**MRI**	**US**	**Other modalities**
Show (0) vs. no-show (1)	0: 71700,	0: 22632,	0: 45181,	0: 3887,
	1: 2687	1: 843	1: 1694	1: 150
	(Total: 74,387)	(23,475)	(46,875)	(4,037)
Not late but wait for more than 1 h	0: 57622,	0: 18124,	0: 36394,	0: 3104,
	1: 13384	1: 4256	1: 8367	1: 761
	(Total: 71,006)	(22,380)	(44,761)	(3,865)

The endpoints include (a) no-show, which means patients who do not show up for their appointment without any notice and (b) long waiting room time, which defines patients who are not late for their appointment but have to wait for more than 1 h.

### 2.2. Statistical analysis

We implemented univariant LR models (R v. 1.3.1093) to find the odds ratio of significant features with each endpoint: no-show and long waiting room times. We split the dataset into training (75%) and test (25%) sets. To define the features with the most significant effects on the endpoints, we implemented the LR using Generalized Linear Models^2^ (GLM) on the training sets.

**Figure 2:**
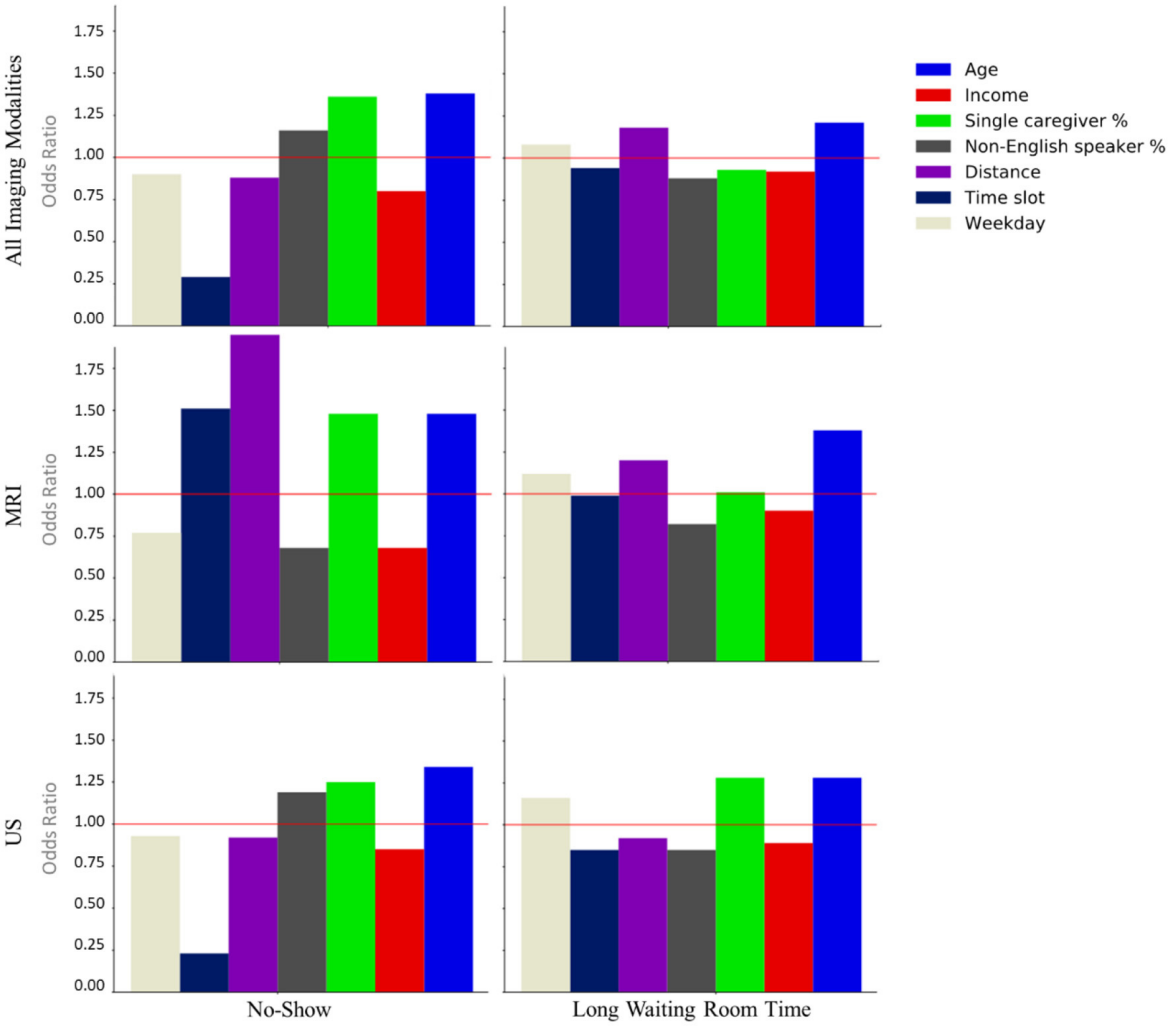
Odds ratios of the statistically significant features (*p* < 0.05) associated with the no-show and long waiting room time endpoints.

Features with *p*-value> 0.05 in the training set were removed. Next, using the remaining significant features from the test set, another LR model was developed and features with *p*-value > 0.05 in the test set were also filtered. The odd ratios for features that remained significant in both training and test sets were calculated. Figure 2 illustrates the odds ratios for the endpoints subjected to all imaging modalities, MRI and US.

#### 2.2.1. Binning

In order to apply machine learning algorithms, optimal binning was used to categorize continuous features (e.g., age). Introduced by Fayyad (40), optimal binning is a discretization method for continuous variables, which is based on Minimum Description Length (41), where patterns in data are utilized to compress the data. Optimal binning fits best if there is a non-linear relationship between the feature and outcome, the bins are more relevant, the original continuous data is extremely noisy, and if the process of binning can be applied to the future data.

We utilized the optbinning python library (42) and our optimal binning was optimized on our training set and then applied to our test set. Appendix A highlightes the settings of Minimum Description Length Principle (MDLP) used for dering the optimal bins for each continuous variable, where “min_sample_leaf” is the minimum number of samples required to be at leaf node, “max_candidates” the maximum number of split points to evaluate at each partition and “min_sample_split” is the minimum number of samples required to split an internal node.

### 2.3. Machine learning

#### 2.3.1. Random forest model

In order to eliminate bias in the appointment scheduling systems, predictive models are needed. Tree-based models are among the common classifiers for tabular data, and RF is ensemble of decision trees, which is utilized in similar contexts such as radiomics (43–45). More gradient boosting-based models, such as XGBoost and NNs are alternatives to RF, that demand more computational resources (46). In this project, we choose RF because it is a reliable classifier as a baseline with affordable computational cost. To conduct the predictive module of the research, we further split the training data into train and validation sets with a ratio of 75/25. The same test cohort used for the statistical analysis was utilized for the final validation. We utilized all features to train a RF model to predict these endpoints. Appendix B contains the selected parameters achieved by a grid search on the training set, which returns the highest F1 score on the validation set. The grid space was set based on the best practices from the literature (43, 47). We considered using the F1 score as the main score for the grid search since it combines the precision and recall and shows how relevant the perditions are to the actual outcome. To tackle the data imbalance, we used the RF's built-in function from Scikit-Learn^3^ to manipulate the weight of each class by adjusting each class's weight, inversely proportional to the class frequency.

#### 2.3.2. Evaluation

We tested the model with the selected parameters on the validation set and defined the optimum threshold for the final prediction on the test set using Geometric Mean or G-mean. This metric is used in imbalanced classification to find a prediction threshold that maximizes both precision and recall. To find the optimal threshold, we have to maximize the G-Mean with regards to the threshold as in Equations (1–3):


(1) $$\begin{array}{l} Sensitivity=True\;Positive\;Rate=\;\frac{T_{P}}{T_{p}+F_{N}} \end{array}$$

(2) $$\begin{array}{l} Specificity=\;1-False\;Positive\;Rate=\;\frac{T_{N}}{T_{N}+F_{P}} \end{array}$$

(3) $$\begin{array}{l} G-Mean=\;\sqrt{Sensitivity\;\times \;Specificity} \end{array}$$

## 3. Results

### 3.1. Statistical analysis results

Our LR analysis showed sex was not a predictor of the endpoints (*p*-value > 0.05). Table 3 shows odds ratios of the significant features (*p*-values < 0.05) and their corresponding endpoints for the three different training and test sets. Our results show families are less likely to miss an appointment (no-show) if they have an evening appointment (OR = 0.29), have higher household income (OR = 0.80), or live farther from the hospital (OR = 0.88). Conversely, families are more likely to miss an appointment if the patient is older (OR = 1.38), they are coming from a postal code with a higher percentage of single caregivers (OR = 1.36), or non-English speakers (OR = 1.16). Surprisingly, no-show endpoints is more frequent when the patients live closer to the hospital.

**Table 3:** Odds ratios of the most significant features.

**Dataset**	**Event**	**Feature**	**Odds-ratio**	**Feature**	**Odds-ratio**
All (whole dataset)	No-show	**Age**	1.38	Distance	0.88
All (whole dataset)	No-show	Single caregiver	1.36	Income	0.80
All (whole dataset)	No-show	Non-English	1.16	**Encoded time slot**	0.29
MRI	No-show	**Single caregiver**	1.95	Distance	0.77
MRI	No-show	Non-English	1.51	Income	0.68
MRI	No-show	Age	1.48	**Encoded time slot**	0.22
US	No-show	**Age**	1.34	Distance	0.92
US	No-show	Single caregiver	1.25	Income	0.85
US	No-show	Non-English	1.19	**Encoded time slot**	0.23
All (whole dataset)	Waiting time	**Age**	1.21	Single caregiver	0.93
All (whole dataset)	Waiting time	Distance	1.18	Income	0.92
All (whole dataset)	Waiting time	Day of the week	1.08	**Non-English**	0.88
MRI	Waiting time	**Age**	1.38	Encoded time slot	0.99
MRI	Waiting time	Distance	1.20	Income	0.90
MRI	Waiting time	Day of the week	1.12	**Non-English**	0.82
US	Waiting time	**Age**	1.28	Single caregiver	0.92
US	Waiting time	Distance	1.16	Income	0.89
US	Waiting time	Day of the week	1.09	**Non-English**	0.85

Bold values refer to features with highest and lowest odds-ratios for different endpoints and datasets (US, MRI, All).

Patients are less likely to wait for more than 1 h for their appointments if their postal code is associated with a higher percentage of non-English speakers (OR = 0.88), a higher household income (OR = 0.92), or higher percentage of single-care givers (OR = 0.93). Older patients (OR = 1.21), and those who live farther from the hospital (OR = 1.18) tend to wait longer for their appointments.

### 3.2. Random forest model results

Table 4 shows the results of the RF model as well as the corresponding feature importances. The upper section of the table includes the performance of the predictive classifiers, and the lower section provides features' importance of the models. Separate classifiers were trained to predict no-show and long waiting room time, and thus their performance evaluation and feature importances are provided in separate sections. Additionally, we had distinct classifiers trained on department schedules for MRI, US appointments, and the whole dataset (MRI and US), whose performance and features' importance are reported in separate rows. We highlight the most important feature (e.g., Time slot for No-show) with a bold font. We could achieve AUC and recall of 0.82 in no-show prediction with MRI patients and AUC of 0.73 in long waiting room time prediction for all patients. In no-show prediction, despite the reasonable AUC and recall, the precision and F1 score were low (0.16 and 0.09, respectively).

**Table 4:** Results for the predictive RF model.

	**Dataset**	**F1 score**	**Precision**	**Recall**	**AUC**	**Accuracy**
	**No-show**					
**Model performance**	**MRI**	**0.16**	**0.09**	**0.82**	**0.82**	**0.70**
	**US**	**0.15**	**0.08**	**0.81**	**0.81**	**0.68**
	**All**	**0.16**	**0.09**	**0.77**	**0.80**	**0.69**
	**Long waiting room time**					
	**MRI**	**0.41**	**0.31**	**0.60**	**0.67**	**0.71**
	**US**	**0.40**	**0.29**	**0.64**	**0.71**	**0.65**
	**All**	**0.43**	**0.31**	**0.66**	**0.73**	**0.66**

	**Dataset**	**Income**	**Distance**	**% Non-English speaker**	**% Single caregiver**	**Age**	**Time slot**	**Sex**	**Weekday**
	**No-show**								
	MRI	0.089	0.082	0.085	0.089	0.132	**0.452**	0.012	0.057
Feature importance	US	0.077	0.080	0.079	0.080	0.132	**0.472**	0.013	0.066
	All	0.077	0.076	0.074	0.077	0.218	**0.370**	0.026	0.084
	**Long waiting room time**								
	MRI	0.113	0.139	0.120	0.111	**0.309**	0.109	0.019	0.080
	US	0.104	0.132	0.106	0.103	**0.326**	0.124	0.017	0.088
	All	0.103	0.118	0.106	0.102	**0.362**	0.073	0.029	0.108

Bold values in feature importance refer to the most predictive features on different endpoints and datasets (US, MRI, All).

**Figure 3:**
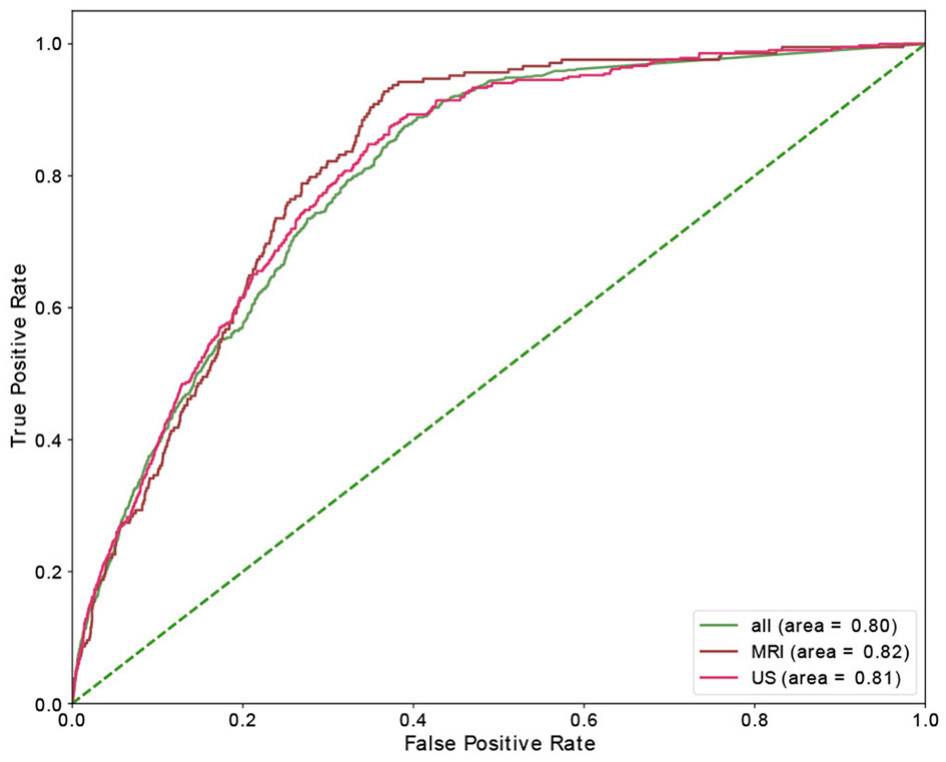
ROC curve of multivariant RF model for no-show endpoint.

**Figure 4:**
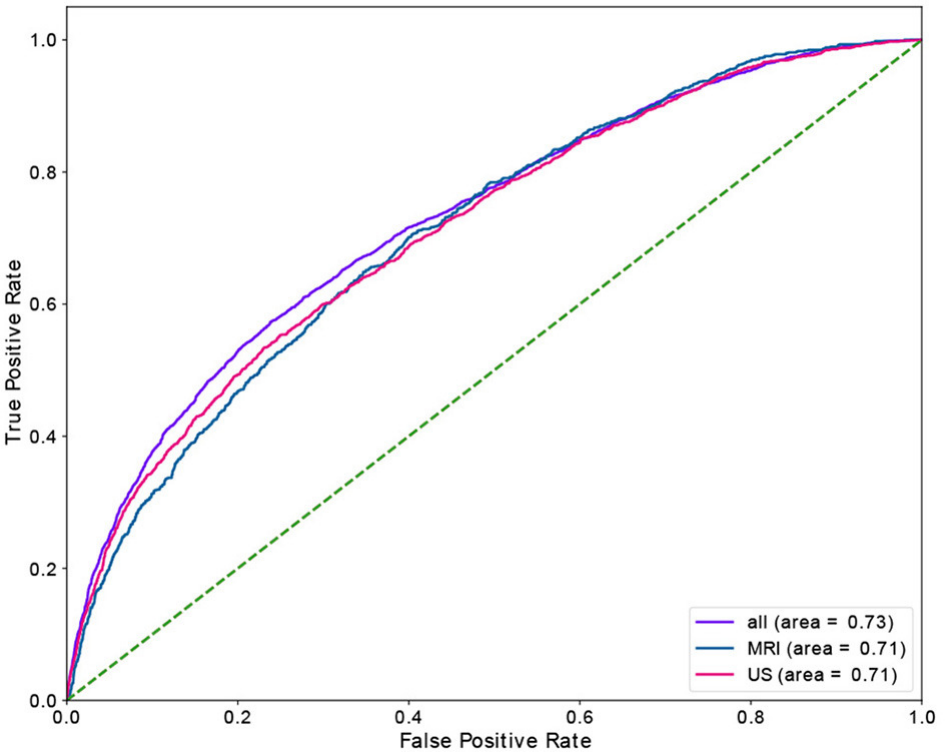
ROC curve of multivariant RF model for waiting-room-time endpoint.

Time slot and age contributed the most for no-show prediction, and age was the most important feature, with sex being the most insignificant feature in the prediction of the long waiting room time. Figures 3, 4 depict the receiver operating characteristic (ROC) curves of the multivariate RF for different datasets and endpoints.

## 4. Discussion

In this applied machine learning research, we investigate whether equity of diagnostic imaging services at our hospital could be improved. Thus, novelty of the research question, complexity of the machine learning classifiers, as well as the classification accuracies of the pipelines are of lesser concerns and will be covered in separate projects where the goal will be to bridge the gap.

To explore the possible trends in access to DI appointments as a function of equity-related patient identifiers, we collected and analyzed appointment data of 42,795 unique patients of the DI department at SickKids Hospital during 2018–2021. We cleansed the dataset, filtered out about 12K rows, and developed 9 different datasets: the training, validation, and test set for three different categories including All, only MRI, and only US patients.

The selected features from the datasets were: age, sex, income, distance, percentage of non-English speakers in a postal code, parentage of single caregivers in a postal code, time slot (morning, afternoon, evening), and day of the week (Monday to Sunday). The endpoints include no-show and waiting room time for more than 1 h.

Our univariate LR analysis on DI appointments revealed that the likelihood of both no-shows and long waiting room time are affected by not only the time of appointment during the day/week, but also patients' demographics such as income and the number of caregivers. Age, time slot, and percentage of single caregiver contributed the most for no-show prediction. Similarly, age, distance, and percentage of non-English speakers had the highest contribution to long waiting room time prediction.

Using a RF model, we achieved AUC of 0.82 and 0.73 for no-show, and long waiting room time endpoints, respectively. The most significant features in RF contributing to the prediction of no-shows were time slot and age. Additionally, for long waiting room time the significant features were age and distance. Sex was an insignificant feature in the prediction of endpoints. While the RF models yielded reasonably AUCs, the precision scores (and consequently the F1 scores) were relatively low. The main reason is the highly imbalanced data where the probability of having a positive class (e.g., no-show) is extremely low (e.g., ~3.6%).

While there were several options to be used in this context, we chose RF, which are among the acceptable algorithms used in recent publications in areas such as radiomics classification (43, 44, 46). Nevertheless, we tested logistic regression and extremely randomized trees (48), and RF was found to be the superior classifier.

The fact that classifier performances are near the ceilings relies on two factors: (a) the acceptable range for the performance of classifiers is subjective (47), and in this context, no classifier will achieve high accuracies because the hospital aims to provide equitable service to the patients and the features are not strongly predictive. Otherwise, it would show systematic inequity and discrimination. (b) there are 8 features, and the order of examples is 10,000. Hence there are multiple cases where the features are identical, but the labels are different. In this situation, even the most advanced models are incapable of classifying all cases correctly. In other words, no model could overfit this training data with ideal accuracy.

While artificial intelligence (AI) can help to minimize the number of no-shows by overbooking for patients who are deemed by AI to miss their appointments, if not done carefully, this can by itself lead to another layer of inequality. Thus, it is imperative to understand and tackle the root causes of inequality, rather than treating it as a resource optimization problem. Our experiment explicitly addresses disparities and inequity in healthcare access experienced by lower socioeconomic status families. Lower-income households tend to have less flexible work schedules, less access to paid time off, and experience greater difficulty commuting to SickKids. Consequently, these families may experience greater hardships around the scheduling of their child's DI appointments.

We note that considerable controversy surrounds the notion of predicting no-shows. Anecdotally, there have been many reports of patients being double-booked for their appointments by algorithmic scheduling tools. These tools are typically guided by a primary value of efficiency (in contrast with equity), wherein the cost savings to the hospital are prioritized, as is the desire to have the highest number of patients seen to minimize wait times. These are reasonable goals; however, in practice, they can result in discrimination as marginalized or disadvantaged patients experience the greatest proportion of inconvenience by virtue of the algorithm. In future work, by centring equity, we will take the same prediction task as prior work but use these patterns to hypothesize on whether no-shows (and long waiting room times) could be minimized by using the predictions to prioritize patients for appointments based on their needs.

This research will lead to proactive and targeted decisions that advance the interests of those who are the least advantaged and are under-represented in healthcare system to provide care in a manner that compensates for the relative lack of privileges that others enjoy. We intend to develop and deploy a model that aims to reduce no-show rates by offering prioritized scheduling for equity-seeking groups in order to achieve a notion of institutional efficacy with respect to DI scheduling and access, while making access easier for our patients and families experiencing structural disadvantage.

This study has limitations. While the proposed models in this study identify inequality in the scheduled appointments, how to achieve equitable appointments remains unanswered. Designing an equitable scheduling system imposes extra limitations such as decision-making with limited available time slots. In addition, this study does not consider the factor of time. Equity should be continuously monitored, and separate test sets should be created and evaluated for different time spans (e.g., years, seasons, months).

In summary, we studied two endpoints at the Diagnostic Imaging (DI) department of Hospital for Sick Children, Canada, Ontario: no-show and long waiting room time. To show the relationship between selected features such as sex, age and socioeconomic status and the endpoints, univariate LR models were applied to ~74,000 appointments. Our analyses show that while no sex discrimination existed among the scheduled pediatric DI appointments, there were inequities based on patient features such as low income and language barrier. Using a RF model, Using a RF model, we achieved AUC of 0.82 and 0.73 for no-show, and long waiting room time endpoints, respectively.

## Data availability statement

Although the institutional research ethics board approval allowed for retrospective data usage without explicit consent, it does not permit data sharing. Researchers wishing to access the data may reach out to the corresponding author to obtain permission under an REB/IRB approved data transfer agreement.

## Ethics statement

The studies involving human participants were reviewed and approved by the Research Ethics Board of Hospital for Sick Children. Written informed consent from the participants' legal guardian/next of kin was not required to participate in this study in accordance with the national legislation and the institutional requirements.

## Author contributions

KN, KK, MM, WL, and FK contributed to the design of the concept and study. KN, MM, WL, and FK contributed to the design of statistical and machine learning modules. KN, MT-S, WL, and KL contributed to data preparation. MT-S, KN, and FK contributed to the implementation of the statistical and machine learning modules. All authors contributed to the writing and reviewing of the manuscript and approved the final manuscript.

## Footnotes

^1^ https://www12.statcan.gc.ca/census-recensement/2016/dp-pd/prof/details/download-telecharger/comp/page_dl-tc.cfm?Lang=E

^2^ https://www.rdocumentation.org/packages/stats/versions/3.6.2/topics/glm

^3^ https://scikit-learn.org/stable/modules/generated/sklearn.ensemble.RandomForestClassifier.html

## Appendix

**Appendix A:** Settings of the optimal binning.

**Min_samples_leaf**	**Max_candidates**	**Min_sample_split**
2	2	32

**Appendix B:** Hyperparameters and settings^*a*^ of the RF models.

**Bin:**	**Continuous variable**
n_estimator:	500
Criterion:	Entropy
Min_samples_split:	10
Max_features:	Auto
Class-weight:	Balanced-subsample

a https://scikit-learn.org/stable/modules/generated/sklearn.ensemble.RandomForestClassifier.html.
